# Assessing the completeness and comparability of outcomes in systematic reviews addressing food security: protocol for a methodological study

**DOI:** 10.1186/s13643-019-1268-1

**Published:** 2020-01-09

**Authors:** Solange Durão, Marianne Visser, Tamara Kredo, Ian J. Saldanha

**Affiliations:** 1 Cochrane South Africa, South African Medical Research Council, Francie Van Zijl Dr, Parow Valley, Cape Town, 7501 South Africa; 2 Centre for Evidence-based Health Care, Department of Interdisciplinary Health, Faculty of Medicine and Health Sciences, Stellenbosch University, Cape Town, 800 South Africa; 3 Center for Evidence Synthesis in Health, Department of Health Services, Policy and Practice (Primary) and Department of Epidemiology (Secondary), Brown University School of Public Health, 121 S Main St, Box G-S121-8, Providence, RI 02903 USA

**Keywords:** Outcome pre-specification, Outcomes reporting bias, Systematic review methods, Food security

## Abstract

**Background:**

Systematic reviews should specify all outcomes at the protocol stage. Pre-specification helps prevent outcome choice from being influenced by knowledge of included study results. Completely specified outcomes comprise five elements: (1) domain (title), (2) specific measurement (technique/instrument), (3) specific metric (data format for analysis), (4) method of aggregation (how group data are summarised), and (5) time points. This study aims to assess the completeness of outcome pre-specification in systematic reviews of interventions to improve food security, specifically food availability, in low- and middle-income countries, as well as to assess the comparability of outcome elements across reviews reporting the same outcome domains.

**Methods:**

We will examine systematic reviews from an ongoing overview of systematic reviews, which assessed the effects of interventions addressing food insecurity through improving food production, access, or utilisation compared with no intervention or a different intervention, on nutrition outcomes. We will examine the original protocols; if unavailable, we will examine the “Methods” section of the systematic reviews’ most recent version. One investigator will identify and group all outcome domains that the authors of the included protocols intended to measure in the systematic review and a second investigator will verify the domains. For outcome domains reported in at least 25% of protocols, one author will extract data using a pre-specified form and a second author will verify the data. We will use descriptive statistics to report the number, types, and degree of specification of outcomes in included protocols. We will assess the extent of completeness of outcome pre-specification based on the number of outcome elements (out of five). We will assess comparability of outcome domains through examining how individual elements are described across SRs reporting the same outcome domains.

**Discussion:**

Our findings will contribute to understanding about the best approach to pre-specify outcomes for systematic reviews and primary research in the field of food security.

## Background

Systematic reviews (SRs) summarise evidence to inform health decision-making. All outcomes important for patients, clinicians, and policy-makers should be pre-specified in SRs to avoid selective reporting of outcomes based on the results in the included studies. Furthermore, completeness of outcome specification may improve the utility of a SR for decision-makers.

In clinical trials, pre-specification of outcomes reduces the number of statistical analyses and thus the risk of type 1 error, i.e. the likelihood of finding a significant effect when there is no true effect. Pre-specification also decreases the risk of outcome reporting bias, i.e. selective or inadequate reporting of outcomes based on the strength or direction of the results. This bias has also been reported in SRs [1, 2]. Pre-specification of outcomes at the protocol stage, i.e. before data collection and analyses have been done, prevents decisions, such as which outcomes, which specific measurements, and which time points should be examined, from being influenced by the knowledge of the results [3]. Although these are common issues in trials, they are also increasingly recognised as being important for SRs [4, 5].

SRs gather data from multiple primary studies, which often report data on the same outcome in different ways; pre-specification prevents SR authors from making post-hoc decisions regarding studies and outcomes for inclusion in the SR [6]. Research has shown that trials assessing the same condition and intervention often report multiple different outcomes as they disagree on the important outcomes to measure, that outcomes selected across reviews on the same condition differ, [6] and that outcomes reported across trials and SRs on same condition have limited overlap [7, 8]. Mayo-Wilson and colleagues [9] found, in a sample of reports addressing the same intervention, that many trials were associated with more than one report and that some outcome domains had multiple definitions, with variations in measures, metrics, etc. Consequently, they found that there were often multiple results that could be associated with the same outcome domain, and thus different meta-analyses using different outcomes could change the magnitude and statistical significance of the meta-analysis and thus the conclusions of a SR. To reduce the risk of outcome reporting bias, it is recommended that outcomes are fully pre-specified in SRs [9, 10].

Cochrane recommends limiting the number of outcomes (ideally to no more than seven) in a SR and pre-specifying them [11]. SR authors should focus on outcomes likely to be most meaningful to clinicians, patients, and policymakers and should interpret cautiously any indirect or surrogate outcomes (e.g. serum albumin for nutritional status). Sometimes, the patient-important and clinically meaningful outcomes for decision-making are not reported in primary studies included in a SR, and this would be an important gap in primary research to highlight at the SR level.

A common framework has been developed and used for defining outcomes and assessing the completeness of their pre-specification [6, 12, 13]. This framework is also now recommended as part of new Cochrane Handbook guidance [10]. According to this framework, a completely specified outcome comprises five elements: (1) *outcome domain* or title, (2) *specific measurement* or technique/instrument used, (3) *specific metric* or format of outcome data that will be used for analysis, (4) *method of aggregation* or how data will be summarised, and (5) *time points* that will be used for analysis (Table 1). This useful and intuitive framework has been used to assess SRs, such as those produced by Cochrane Eyes and Vision [12] and Cochrane Wounds [6] Review Groups, but has not been used to assess SRs of interventions to address food security.

**Table 1:** Nutrition-related examples of the five elements of a completely specified outcome

Element	Example
1) *Outcome domain* or title	Weight
2) *Specific measurement* or technique/instrument used to take the measurement	Digital or balance beam weighing scales
3) *Specific metric* or format of outcome data that will be used for analysis	Value at a time point or change from baseline
4) *Method of aggregation* or how data from each group will be summarised	Mean (standard deviation) *Z*-score
5) *Time points* that will be used for analysis.	Short term (2 months or less), medium term (less than 1 year), and long term (more than 1 year)

Food security exists when people have physical, social, and economic access to sufficient, safe, and nutritious food that meets their dietary needs and food preferences for an active and healthy life [14]. A world without hunger and malnutrition is one of the United Nations Sustainable Development Goals (SDGs) [15]. There is currently a rise in global hunger, particularly in Africa and South America; one in nine people in the world were undernourished in 2017 [14]. Child undernutrition is a major public health burden, especially in low- and middle-income countries (LMICs), where 66% and 75% of all children under the age of 5 years who are stunted and wasted in 2017, respectively, live [16]. Malnutrition resulting from hunger and food insecurity has detrimental consequences for individuals and societies. At the individual level, it leads to poor child development and health, increased risk of disability, morbidity, and mortality, and decreased productivity and income-generating potential in adults who suffered from malnutrition in early childhood [17–19]. At the societal level, malnutrition is associated with direct losses in economic productivity due to poor mental and physical performance and premature death, and with indirect losses due to decreased working and intellectual capacity of the working population, and to loss of resources due to increased health care costs [19]. It is therefore important to address food insecurity, and SRs may be able to identify the most effective interventions.

Through the authors’ experience in conducting an overview of SR (ongoing) and a SR addressing food security-related questions [20], they identified multiplicity of outcome reporting in SRs and primary studies, which makes it difficult to harmonise and synthesise this evidence. Since pre-specification contributes to reducing reporting bias, we felt it was important to assess whether and to what extent authors of SRs addressing food security do this. Through this process, we will also be able to identify commonly reported outcomes across these SRs, which will contribute toward a planned next phase of identifying core outcome sets for food security. The development of core outcome sets is another recommendation for reducing outcome reporting bias in the SR process; it helps ensure that most appropriate review outcomes are selected and reported in primary studies, which will then inform systematic reviews [21]. There has been some work looking at the importance of identifying core indicators for food security, as a more efficient way of measuring and reporting progress toward achieving food security that does not rely on long lists of indicators that are not easily comparable across time and space [22, 23]. However, existing guidance, although informative, is more geared toward country-level monitoring, or does not include all relevant outcome domains, and thus is not very useful to identify core outcomes to report at primary research and SR levels.

Therefore, we aim to use the outcomes definition framework described above to assess the completeness of outcome pre-specification in a set of SRs addressing food security. We also aim to assess the comparability of outcome elements across SRs reporting the same outcome domain. Harmonisation of outcomes across SRs addressing related questions is potentially useful for cases where overviews of SRs are conducted to inform decision-making [11].

## Objectives

This project aims to (1) describe all outcomes reported in SRs of interventions designed to improve food security in low- and middle-income countries (LMICs), (2) assess the completeness of outcome pre-specification in these reviews, and (3) assess the comparability of outcome elements across reviews reporting the same outcome domains. Our findings, once disseminated, can lead to better awareness of the importance of appropriate pre-specification and reporting of outcomes in SRs and primary research in this field. Additionally, our findings can inform a subsequent project aiming to identify core outcomes for this area.

## Methods

### Selection of protocols and SRs

For this study, we will include SRs that are being evaluated in an ongoing overview of SRs of interventions addressing food security. The eligibility criteria are:

- Participants (P)—Any communities or groups of individuals in LMICs, across all life cycle stages;
- Interventions (I)—Any intervention to address food insecurity through improvements in the production, access, or utilisation of food, or a combination of these;
- Comparisons (C)—No intervention or a different intervention to address food insecurity;
- Outcomes (O)—Any outcome related to food security, including nutritional status, measured at national or regional, district or community, or at household and individual levels.
- Study Design (D)—SRs, defined as reviews that had pre-determined objectives, predetermined criteria for eligibility, searched at least two data sources of which one needed to be an electronic database, and performed data extraction and risk of bias assessment [24]. Both protocols and completed systematic reviews were eligible.
- Setting (S)—Included at least one study from a LMIC (according to the World Bank definition; https://datahelpdesk.worldbank.org/knowledgebase/articles/906519-world-bank-country-and-lending-groups).

To identify SRs for the overview, we searched seven electronic databases from 1979 to February 2015: (1) MEDLINE (via PubMed), (2) the *Cochrane Database of Systematic Reviews*, (3) Database of Abstracts of Reviews of Effects (DARE) through the Cochrane Library, (4) Trip Database (which includes records in the EPPI library), (5) www.healthevidence.org, (6) Campbell library, and (7) 3ie database (more details regarding this overview are available on request).

We will seek the original protocols of the included SRs, through searching for links to the protocol in the included review, searching PROSPERO, and searching the Cochrane Library for the protocol version of the review (in the case of Cochrane Reviews). If necessary, we will also contact authors and Cochrane Review Group editors. If the original protocol is not available for a given SR, we will use the “Methods” section of the most recent version of the SR (if it had been updated). For simplicity, we hereafter refer to each SR as a “protocol.”

### Selection of outcome domains

One investigator will identify and group all outcome domains that the authors of the included protocols intended to measure in the SR. To do so, we will review all sections of the protocols, not just the “Methods” sections. A second investigator will verify the definition and grouping of the domains identified. Discrepancies in outcome categorisation will be discussed by the author team. Of all outcome domains identified, we will select the key outcome domains as those that are reported in at least 25% of included protocols for data extraction.

### Data extraction

Data items extracted from each protocol will include:

1. Publication year and first author (if the protocol was published) and date of protocol (if the protocol was not published).
2. Details regarding the intervention category (i.e. addressing food availability, access, or utilisation) and types of intervention within each category (e.g. agricultural interventions, income-generating interventions, cash transfers, nutrition education).
3. Data regarding the five elements of outcome pre-specification for each outcome [6, 12, 13]
  i. Domain,
  ii. Specific measurement used (this element will be considered specified if the SR authors stated how the outcome should be measured, including [where relevant] instruments, tools, scales, scores, and/or how the outcome should be defined),
  iii. Specific metric used (this element will be considered specified if the SR authors specified how they would analyse the data, including change from baseline, value at a time point, or time-to-event),
  iv. Method of aggregation used (this element will be considered specified if the SR authors specified how the data were to be summarised, including as mean, median, percentage or proportion, or an absolute number), and
  v. Time points at which outcomes were measured or analysed (this element will be considered specified if the authors specified the time points to be used in their analysis).

Each outcome domain could have more than one outcome specification, in which case we will extract all specifications. Two authors will pilot the data extraction form using four protocols. Subsequently, one author will conduct data extraction for all protocols, and a second author will verify all extracted data. Any disagreements will be resolved through discussion.

### Data analysis

We will report the number, types, and degree of specification of outcomes in included protocols using descriptive statistics. We will assess the extent of completeness of outcome pre-specification based on the number of outcome elements specified out of five possible elements. We will summarise the median and interquartile range (IQR) for the number of outcome elements specified for each included outcome domain and describe the elements in more detail for each domain. We will also report the frequency of categories of specific measurement, metric, method of aggregation, and time points for each outcome domain. To assess comparability of outcome domains, we will assess how individual elements are described across SRs reporting the same outcome domains.

## Discussion

This study is underway; SRs have been identified and full data extraction will begin.

If any changes are made to the protocol in the final research report, these will be marked clearly and justified. This research will contribute understanding about the pre-specification and reporting of outcomes in SRs addressing food security, which could potentially inform the identification of standard outcome measures that should be reported in this field.

## Data Availability

The datasets used and/or analysed during the current study are available from the corresponding author on request.
